# Dental Polychrests

**Published:** 1864-01

**Authors:** W. H. Atkinson

**Affiliations:** New York


DENTAL POLYCHRESTS.—*By W. H. Atkinson,* M. D.—1. Pure creosote; 2. A saturated solution of resublimed iodine in pure creosote; 3. Officinal tincture of iodine; 4 A saturated solution of pure iodine in Price's glycerin; 5. Officinal wine of opium; 6. Pure chloroform (American, by Squibb, of Brooklyn, and the Scotch are preferable); 7. Camphor gum.

These seven articles, singly and in various combinations, may be made to fulfill almost any indication desired by the dentist.

Dr. Ziegler, of Philadelphia, suggests that various indications may be met by a combination of camphor, iodine, creosote, and chloroform, or substituting for differing conditions, arnica, morphia, or aconite, instead of the chloroform.

Creosote in its pure state acts primarily as a painful escharotic upon denuded or delicate cuticular surfaces, with a secondary soothing, intoxicating, and sometimes blissful reaction very grateful to the patient. No fungous growth can long resist its continued application.

The rule is, to repeat as long as pus is formed on the surface or prurient molecules spring up, but never repeat so long as the eschar of the former application remains attached.

In some organizations the epithelium exfoliates in twelve hours, while in others it requires forty-eight hours or more to desquamate the dead pellicle.

Iodine in creasote acts much like the above, but is more penetrating, making a deeper eschar and more rapidly polarizing (hardening) the new granulations, the growth of which it has the power to hasten when not too abundantly applied. It is better adapted to the more malignant cases than creosote alone.

Tincture of iodine may also be made to cause slough if used in sufficient quantity and kept pressed upon the part, but its principal use is as a stimulator to granulation and a hardener of the molecules to the standard of health normal to the part.

The glycerole of iodine is better adapted to the more benign forms of apthæ and gum boil when very circumscribed and mild.

Wine of opium is a good soothing stimulus to weakened mucous membranes or newly-formed plasm out of which we desire to generate the new part. Used generally after or in alternation with simple tincture of iodine after pus has been nearly or quite prevented from forming.

Pure chloroform is a powerful stimulus, and if confined closely upon the part is escharotic to some extent on weak tissues.

Camphor, as is well known, is almost a universal antidote or modifier, in greater or less degree, of the actions of other drugs. Its especial use to the dentist is best displayed in its ability to deodorize creosote, in which it is very soluble. About twenty grains to the ounce is capable of covering the peculiar smell of creosote which is so very objectionable to some persons. It is a great modifier of the escharotic character of creosote without impairing its antiseptic qualities as an application to all cavities before filling with gold or other material, which has so signally blessed all those who have resorted to it for this purpose. Thus used, it enables the dentist to preserve a stratum of decalcified dentine as a cap to protect the pulp with perfect impunity.

The very general prejudice against creosote, iodine, and camphor arises from ignorance of their properties and the best way to use them. These, as well as all other remedies, should be used with direct reference to the patient's good rather than to our convenience, and as delicately and neatly as possible.

He who uses an instrument or a remedy in a bungling and awkward manner, proves his lack of fitness for his place more than his lack of knowlodge how best to administer to his own personal interests and advancement. Even a blunder handsomely and delicately apologized for will go further to advance a man's usefulness and reputation than the deepest erudition harshly and coarsely announced and put in practice. First be sure that all your purposes are clean, then be careful to prepare yourself to administer your polychrests in the most, attractive and intelligent manner; and my word for it, you will never have cause to discard nor limit their range of usefulness as the nature of tneir adaptability increasingly unfolds to your improved power of perception of adaptation of means to ends.

NEW YORK, Dec. 22, 1863.—*Dental Cosmos.*

